# *Lactobacillus*, glycans and drivers of health in the vaginal microbiome

**DOI:** 10.20517/mrr.2022.03

**Published:** 2022-05-13

**Authors:** Rosemary Sanozky-Dawes, Rodolphe Barrangou

**Affiliations:** Department of Food, Bioprocessing and Nutrition Sciences, North Carolina State University, Raleigh, NC 27606, USA.

**Keywords:** Glycans, vaginal, microbiome, *Lactobacillus*, immunity

## Abstract

A microbiome consists of microbes and their genomes, encompassing bacteria, viruses, fungi, protozoa, archaea, and eukaryotes. These elements interact dynamically in the specific environment in which they reside and evolve. In the past decade, studies of various microbiomes have been prevalent in the scientific literature, accounting for the shift from culture-dependent to culture-independent identification of microbes using new high-throughput sequencing technologies that decipher their composition and sometimes provide insights into their functions. Despite tremendous advances in understanding the gut microbiome, relatively little attention has been devoted to the vaginal environment, notably regarding the ubiquity and diversity of glycans which denote the significant role they play in the maintenance of homeostasis. Hopefully, emerging technologies will aid in the determination of what is a healthy vaginal microbiome, and provide insights into the roles of *Lactobacillus*, glycans and microbiome-related drivers of health and disease.

## THE VAGINAL MICROBIOME

The human vaginal microbiome comprises a diverse set of organisms that can associate with health or disease and vary across populations^[1-3]^. It is a complex ecosystem, varying over the course of a woman’s life, constantly fluctuating during the menstrual cycle^[4]^. Historically, five community state types (CSTs) have been used to describe the vaginal microbiome, encompassing four *Lactobacillus*-dominant communities that primarily consist of *L. crispatus*, *L. gasseri*, *L. jensenii*, and *L. iners*, and one non-*Lactobacillus* dominant diverse community^[5]^. Altogether, the *Lactobacillus*-dominated groups occur in approximately 70% of women^[6]^. The non-*Lactobacillus* dominant CST typically comprises *Gardnerella*, *Prevotella*, *Sneathia*, *Atopobium*, *Molibuncus*, *Clostridium*, *Corynebacterium*, *Staphylococcus*, *Streptococcus*, *Enterococcus*, and *Mycoplasma*^[3,7-10]^. Although a diversity of gut microbes is typically associated with health, it is not the case in vaginal microbiomes. Indeed, most vaginal microbial populations are dominated by a single genus, *Lactobacillus*, characterized by Gram-positive anaerobic or microaerophilic rods with peptidoglycan cell walls^[11]^. This genus is commonly associated with better clinical outcomes^[12]^. Although diverse *Lactobacillus* species are associated with a healthy vaginal microbiome, they all usually produce D- and L-lactic acid, which is inhibitory to pathogens and creates anti-inflammatory conditions^[13-15]^. However, some species like *L. iners* produce moderate amounts of L-lactic acid, but do not produce the D-isomer^[14-16]^. Besides their biochemical attributes, structural elements on the bacterial surface also contribute to the microbe-host molecular dialogue^[17]^. Indeed, microbial recognition hinges on the presentation of cell surface components, especially glycans that interact with host epithelial and immune cells^[17-20]^. The surface composition differs across species such as *L. gasseri*, *L. jensenii*, *L. iners* and *L. crispatus*, and others, notably with regard to S-layers. Of note, *L. crispatus* is the only characterized vaginal *Lactobacillus* that produces an S-layer, which is comprised of non-covalently bound “crystalline arrays of self-assembling proteins found outermost on the cell wall”^[11]^.

## SURFACE COMPONENTS

The S-layer and S-layer-associated proteins (SLAPs) of *Lactobacillus acidophilus* have been studied and characterized most extensively amongst lactobacilli^[11,21]^. Such S-layers are associated with cell shape, enhanced adherence to host epithelial cells, and immunomodulatory responses^[11,22]^. Dendritic cells (DCs) are involved in molecular pattern recognition through sensors like toll-like receptors (TLRs). Studies involving S-layer proteins of the widely commercialized probiotic strain *L. acidophilus* NCFM^[23]^ have implicated SlpA, SlpB, and SlpX in immunomodulation. In particular, SlpA has been shown to interact with receptors on antigen-presenting DCs, which are important sentinels for mucosal surfaces^[24]^. Likewise, the interaction between NCFM and DC-SIGN (Dendritic Cell-Specific ICAM-3 intercellular adhesion molecule grabbing non-integrin)^[17]^ receptor drives the production of anti-inflammatory IL-10, whereas the interaction of an NCFM mutant with a chromosomal inversion over-expressing *slpB* and under-expressing *slpA* led to the production of proinflammatory cytokines^[24]^. The homeostatic anti-inflammatory properties of SlpA were shown to mitigate murine colitis^[25]^ and also act via DCs to trigger signaling pathways that inhibit viral infections^[26]^.

The S-layer of *L. acidophilus* provides a scaffold for numerous SLAPs that are secreted, non-covalently bound^[21]^, and display important surface features^[11,27]^. Hymes *et al.*^[28]^ characterized a SLAP binding to fibronectin, and Johnson and Klaenhammer^[29]^ described another SLAP, the AcmB autolysin, which is involved with *in vitro* binding to mucin and the extracellular matrix proteins fibronectin, collagen, and laminin. The extracellular matrix is a network of molecules produced by resident cells, providing structural support for cells and tissues^[30]^ and regulating cell signaling and adhesion^[31]^. There are also reports exploring the S-layer and S-layer associated proteins of *Lactobacillus crispatus*. Antikainen *et al.*^[32-34]^ demonstrated that S-layer proteins of *L. crispatus* adhere to collagen, and laminin in the context of intestinal cells. An S-layer producing vaginal *L. crispatus *isolate was highly adherent to cervicovaginal epithelial cells and was antagonistic to pathogens of the genitourinary tract^[35,36]^. *In silico* analyses showed the presence of AcmB orthologs in other S-layer-producing *Lactobacillus*^[29]^. When comparing *L. crispatus *genomes, Pan *et al.*^[37]^ found heterogeneity regarding the presence of autolysin and *acmB*-type genes in isolates, though no clear association with a particular isolation source was observed. Furthermore, Tytgat and Lebeer^[38]^ highlight that bacterial glycoconjugates at the microbial surface, including S-layers, may be glycosylated. In fact, microbial glycans comprise much of the bacterial cell surface^[39]^. To date, S-layer glycans have only been confirmed in *L. buchneri* and *L. kefir*^[40]^. Thus, future studies should determine whether *L. acidophilus* and other *Lactobacillus* S-layer proteins are glycosylated^[41]^.

## GLYCOSYLATION AND THE GLYCOME

The glycome is the entirety of a cell’s carbohydrates, either free or as moieties of glycoconjugated macromolecules. Almost all cells are surrounded by glycans, forming a “sugar jacket” comprising proteoglycans, glycosphingolipids, and glycoproteins that form a glycocalyx^[42]^. In vertebrates, mucosal glycan chains typically terminate in various sialic acid molecules^[43,44]^. Within the glycome, the negatively charged sialome^[45]^ plays a role in signaling by concealing antigens on cell surfaces, which consequently appear as “self”, thereby weakening immunoreactivity^[46]^. The diverse functions of glycans include structural modularity with various glycoconjugates, and providing specificity for glycan-binding proteins and receptors^[30,42,47]^. Both receptors and ligands may contain essential glycan domains, such as pattern-recognition receptors, encompassing TLRs which are transmembrane glycoproteins that have evolved to recognize conserved molecular patterns on microbial surfaces, typically referred to as MAMPs (Microorganism-Associated Microbial Patterns) that may also contain glycans^[17,18,48]^. Glycosylation is an essential regulatory mechanism for post-translational processing, which plays a crucial role in the assignment of protein structure, function, and stability, especially 3-D conformation. This in turn influences protein-protein interactions like signaling^[42]^ as well as eukaryotic viral and bacteriophage attachment^[49]^. Some Interleukins, cytokines, viral coat proteins, and G protein-coupled receptors are glycosylated, as well as immunoglobulins and hormones such as gonadotropins, luteinizing hormone, follicle-stimulating hormone, and thyroid-stimulating hormone^[50-52]^. “Essentially all surface-localized immune receptors are glycoproteins”^[53]^. Glycans are integral for immune system modulation and interaction with cells such as macrophages, monocytes, natural killer cells, antigen-presenting cells like dendritic cells, and T cells. These interactions impact both innate and adaptive immunities, cytokine production, and epithelial cell responses^[18,53]^. Glycans can exert dual immune roles, acting either in an inhibitory or stimulatory manner^[18,19]^, and can be involved in tolerance or autoimmunity^[18-20]^.

On the host side, the vaginal mucus is also highly glycosylated. It is composed of glycoprotein mucins, other secreted proteins like immunoglobulins^[54]^, and antimicrobial peptides produced by mucosal epithelial cells and neutrophils, some of which bind glycans^[55,56]^. The branched carbohydrate moieties can make up to 80% of mucin weight^[54] ^and usually terminate in an outermost sialic acid residue^[57]^. Mucus sialoglycoproteins are believed to entrap microorganisms, protecting epithelial cells against infection. Cell surface mucins can bind pathogens, indicating a role of mucus in the innate immunity of the female genital tract^[12,57,58]^. Consequently, the glycome status of host cells, commensal microbes, and mucosal surfaces in a microbiome are important in the maintenance of homeostasis and health, implying that glycome disruption could lead to dysbiosis.

## BACTERIAL VAGINOSIS

In the context of women’s health, bacterial vaginosis (BV) is a prevalent form of vaginal dysbiosis associated with a variety of adverse health outcomes^[59]^. It is often found in non-*Lactobacillus*-dominated microbiomes^[3,4,10]^. Biofilm presence on vaginal epithelial cells is an important factor in the evolution of BV and explains episodes of recurrent infections^[60]^. A signature of BV is the presence of a dense polymicrobial biofilm on the vaginal surface believed to be initiated by *Gardnerella vaginalis*, providing a scaffold for other species to adhere to^[10]^. An under-reported factor in biofilm formation is glycosyltransferase activity, as well as the destructive action of sialidases on the protective mucosal glycan surfaces of vaginal epithelia, which possibly facilitates adhesion of vaginal pathogens such as *Gardnerella* and *Prevotella*^[61,62]^.

Moncla *et al.*^[54]^ have shown that glycosidase and sialidase activity is associated with a reduced number of sialic acid binding sites, a virulence factor in pathogens of mucosal surfaces, and a feature of BV^[58]^. Two bacteria typically associated with BV are *Gardnerella vaginalis* and *Prevotella bivia*. Sialidase is produced by many *P. bivia* isolates, but only by 25% of *G. vaginalis* isolates. The sialidase produced by *Prevotella* is cell-bound, whereas *Gardnerella* sialidases are extracellular and affect the vaginal environment differently^[54,58,62]^. When sialidases are secreted, they may remove sialic acid residues from carbohydrate chains distant from the organism^[63]^, and subsequently affect the function of cells and molecules like immunoglobulins. This would make sialic acids available to other organisms capable of their catabolism and furthermore, expose the “open” carbohydrate chains to exo- and endo-glycosidase attack as well as other hydrolytic enzymes like mucinases, sulfatases, proline dipeptidases, and fucosidases^[19,58,64-66]^. Contributors to *Essentials of Glycobiology*^[67]^, relate fascinating abilities of some pathogens to produce sialidases that “steal” sialic acids from the periphery of host cell glycans to add to their surface for use as immuno-camouflage. For example, some *Neisseria gonorrhoeae* have efficient sialidases that enable this mimicry^[44,67]^. These enzymes may be responsible for altering the vaginal and cervical glycomes, “disrupting dynamic systems responding to internal signals like hormones and to other signals from members of the vaginal microbiome”^[58]^. Furthermore, Moncla *et al.*^[54,58]^ demonstrated this disruption by evaluating the glycome of cervicovaginal lavage and cervicovaginal fluid samples from women with BV *vs. *healthy women. In both BV sample types, they report increased activity of distinct glycosidases such as sialidase, α-galactosidase, β-galactosidase, and α-glucosidase, which are associated with decreased sialic acid binding sites and mannose-binding sites^[54]^. The measurement of binding sites utilized lectins, which are proteins that bind sugar moieties of molecules and are so specific that frequently isomeric glycans with identical sugar content can be distinguished^[68]^. Lectins are found in humans, animals, plants, lichens, bacteria, and higher fungi, and have roles in “cell-cell interactions, signaling pathways, cell development, and immune responses”^[69]^. In the review of Vagios and Mitchell^[12]^, an argument is presented for more research focusing on how mucins and glycans influence vaginal colonization and affect host-microbe interactions, though there is a general paucity of studies investigating the glycome^[54,58,70]^.

## HOST-MICROBE INTERACTIONS

In their insightful opinion, Tytgat and de Vos^[17]^ equate the “array of glycoconjugates on bacterial surfaces as strain-specific barcodes generating diversity as ligands for shaping microbial-host interactions”^[17]^. Even though the field of bacterial glycobiology is expanding, the scarcity of studies on bacterial cell surface protein glycosylation in general, and in the context of women’s health is perplexing. Sun *et al.*^[71]^ carried out a comparative genomic analysis of 213 lactobacilli, and highlighted the diversity of glycotransferases documented. However, surface glycoconjugates cannot be inferred genetically, since they are post-translational modifications, highlighting the need for functional and biochemical characterization^[23]^. Likewise, genomic studies of *L. gasseri*^[72]^ and *L. jensenii*^[73]^ have not substantiated their beneficial roles in the vaginal microbiome. Petrova *et al.*^[6]^ report that *L. jensenii* can reduce adherence and invasiveness of *N. gonorrheae*, and that *L. gasseri* can displace the gonorrhea coccus, but these traits can vary across strains. A characteristic of some *L .crispatus* and *L. gasseri* strains is the presence of genes encoding mucin binding proteins, but additional functional and mechanistic insights are needed.^[12]^ The role of *L. iners* in contributing to vaginal health or disease is unclear and sometimes subject to controversy^[66]^. Indeed, *L. iners* shares attributes with *Gardnerella*, a pathogen associated with BV, such as: a small genome indicative of symbiotic/parasitic lifestyle, moderate lactic acid production^[6,14,15]^, secretion of a cholesterol-dependent cytolysin, and overgrowth during menstruation^[10,16]^. Some believe that *L. iners* may be a transitional organism between health and dysbiosis^[10,74]^. Actually, *L. iners*, is found in low to moderate abundance in the non-*Lactobacillus*-dominated vaginal microbiome^[5,65]^.

A bacterial surface glycoconjugate barcode sends a molecular message to other community members^[17]^. A critical step in deciphering these barcoded messages is to determine the glycosylation of surface proteins in vaginal lactobacilli of interest. This can be achieved by cell shaving with trypsin^[75]^ or by Lithium chloride extraction of S-layer proteins and SLAPs^[11,27]^ and determination of glycosylation using lectin microarrays, monoclonal antibodies, synthetic glycans, or prediction tools^[39,74,76,77]^. In particular, it would be interesting to determine *L. crispatus* surface glycosylation given the association of this species with vaginal health and homeostasis^[78]^. Besides, *L. crispatus* isolates from vaginal, intestinal, and poultry sources display very different S-layer and SLAP profiles, providing a unique opportunity to determine glycome diversity across environments for one species^[21]^.

Glycogen is a major carbohydrate source due to its cyclical release from vaginal epithelial cells. Many commensals rely on host amylases to break it down into usable sugars. Van der Veer *et al.*^[79] ^2019 found some *L. crispatus* isolates with intact pullulanase type 1 genes, enabling them to directly utilize glycogen, which would provide a competitive advantage. Genomes of *L. crispatus* encode diverse hypervariable content, including prophages, autolysins, bacteriocins, and various systems related to mobile genetic elements such as plasmid stabilization systems, toxin-antitoxin systems, and CRISPR (Clustered Regularly Interspaced Short Palindromic Repeat) and associated sequences CRISPR-Cas immune systems^[37,80]^. Studies have reported genomic islands encoding enzymes involved in exopolysaccharide (EPS) production on plasmids in *L. crispatus*, which could enhance adherence, biofilm formation, and exclusion of pathogens^[79]^. Yet, it is unclear how the immunological *L. crispatus* “message” differs between S-layer presentation and EPS, and whether *L. crispatus* alters its surface glycome via EPS. It would be illuminating to determine the glycome of all major vaginal microbiome bacteria. Since many components of extracellular matrices are glycosylated, the ECM of the vaginal microbiome should be explored^[31,81]^. Importantly, the effects of other factors in play should also be determined, notably the virome^[49]^ mycobiome^[82]^, hormones^[13,58]^, metabolites^[7,83]^, stress^[14]^, male factor transfers^[3]^, sexual partners^[6]^, douching^[58,84]^, and endocrine disruptors^[85]^. Unfortunately, since the human cervico-vaginal environment possesses unique attributes such as a particularly low pH, due to *Lactobacillus* lactic acid production^[6,15]^, there is no adequate animal model. Nevertheless, we could exploit rising technologies such as “organ-on-a-chip”^[86]^ or 3D EpiVaginal tissue^TM^^[87]^ for future studies.

## CONCLUSIONS

Despite tremendous advances in the study of the intestinal microbiome, the relative paucity of studies on the vaginal microbiome is puzzling. A deeper and more comprehensive understanding of microbial dynamics and bacterial functions in the vaginal microbiome would drive the development of novel products to maintain, enhance or restore vaginal health and prevent or treat dysbiosis. This could also enable the development of biomarkers to detect microbiome aberrations and diagnose unhealthy conditions^[6]^. Historically, our limited understanding has also been hampered by the lack of glycoscience-related tools, though recent efforts are encouraging, promoted by the Consortium of Functional Glycomics and international entities such as EuroCarb and the Japanese Consortium for Glycobiology and Glycotechnology^[53]^.

**Figure 1:**
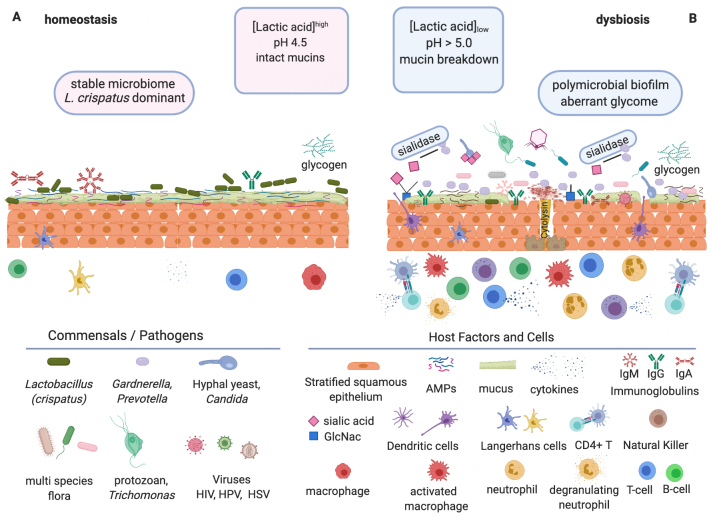
Vaginal homeostasis *vs.* dysbiosis. Glycobiology is key to understanding interactions within the vaginal microbiome since many elements encompassing the microbiota and the host are glycosylated or bind glycans. The immune state is affected^[90]^ in different ways between a healthy state of homeostasis (A) and a disease state of symbiosis, which in turn contributes to either health (A) or dysbiosis (B) characterized by distinct commensals and pathogens interacting with host factors and cells. (A) Homeostasis. *Lactobacillus crispatus *is deemed to be the preferred vaginal microbiome commensal when dominant due to its high lactic acid production, from glycogen degradation, resulting in beneficial low pH. (B) Dysbiosis. This condition does not have the beneficial protective effects of low pH. Presented by multi-species, non-*Lactobacillus *flora, including pathogens such as *Prevotella *and *Gardnerella. *Virulence factors are produced such as: biofilms, hydrolytic enzymes (e.g., sialidases), and cytolysins which can lead to the breakdown of mucins and epithelial cells, disruption of the homeostatic glycome, and immune response (e.g., deglycosylation of immunoglobulins and activation of immune factors). These conditions in turn promote the rise of undesirable members of the microbiome, such as viruses, yeast, and even protozoa^[6,91]^. Figure created using BioRender.com.

McKitrick *et al.*^[39]^ summarized topics covered at a recent NIH workshop entitled “Glycoscience and Immunology at the Crossroads of Biology.” They present a Venn diagram where immunology, microbiology, and glycobiology overlap to encompass glycoscience, infection, and immunity. Participants discussed the need to determine glycan structures, linkages, stereochemical orientation, and functionality^[39]^, as widespread essential factors with variable chain length, linkage, and branching with inherent functional differences^[88]^. Given the implication of glycans in cell activation, differentiation, and development, a deeper understanding of their role in women’s health would be beneficial^[42,89]^. Glycome studies will complement genomic and functional analyses of the vaginal microbiome [Figure 1] and reveal the importance of glycans in other microbiomes. This will open new avenues to manipulate the composition and function of key bacterial species driving women’s health and disease.

## DECLARATIONS

### Copyright

© The Author(s) 2022.
